# Dual SIRT1 expression patterns strongly suggests its bivalent role in human breast cancer

**DOI:** 10.18632/oncotarget.23006

**Published:** 2017-12-06

**Authors:** Khaldoun Rifaï, Gaëlle Judes, Mouhamed Idrissou, Marine Daures, Yves-Jean Bignon, Frédérique Penault-Llorca, Dominique Bernard-Gallon

**Affiliations:** ^1^ Centre Jean Perrin, Department of Oncogenetics–CBRV, 63001 Clermont-Ferrand, France; ^2^ INSERM U 1240–IMoST, 63005 Clermont-Ferrand, France; ^3^ Centre Jean Perrin, Department of Biopathology, 63011 Clermont-Ferrand, France

**Keywords:** breast cancer, molecular subtypes, SIRT1, expression levels, statistical analysis

## Abstract

Breast cancer is the most common cancer in women, and the leading cause of cancer death in women worldwide. SIRT1 (silent mating type information regulation 2 homolog) 1 is a class-III histone deacetylase involved in apoptosis regulation, DNA repair and tumorigenesis. However, its role in breast carcinoma remains controversial, as both tumor-suppressive and tumor-promoting functions have been reported. Also, there are very few reports available where expression of SIRT1 is comprehensively analyzed in breast tumors classified by molecular subtype. Here, using a cohort of 50 human breast tumors and their matched normal tissues, we investigated SIRT1 expression levels in the 5 molecular subtypes of breast cancer according to the St Gallen classification (2013). Tumors and their corresponding normal tissue samples were collected from all patients, and SIRT1 mRNA and protein expression levels were then examined by real-time quantitative polymerase chain reaction and immunoblotting, respectively. After statistical analysis, the results showed a dual expression profile of SIRT1 in human breast carcinoma, with significant overexpression in luminal and HER2-enriched subtypes and significantly reduced expression in the triple-negative subtype. We also found an inverse correlation between SIRT1 expression and breast cancer aggressivity. These novel findings suggest that SIRT1 plays a dual role in breast tumors depending on its expression rate and the molecular subtype of the cancer. Our data also point to a potential role for SIRT1 as a prognostic biomarker in breast cancer.

## INTRODUCTION

Breast cancer is the most common cancer in women, and the leading cause of cancer death in women worldwide [1]. It is a multifactorial genetic disease with different prognoses for different subtypes. According to the St Gallen breast cancer classification [2], there are five distinct molecular subtypes of breast cancer classified in ascending order of tumor aggressiveness, from luminal A, relatively the least aggressive with the most favorable prognosis and survival rate [3], to luminal B (HER2-) and luminal B (HER2+), these 3 subtypes are included in the Hormone Receptor-positive Breast Cancer (HRBC), HER2-enriched or HER2 Breast Cancer (H2BC), and finally triple-negative breast cancer (TNBC), also known as basal-like, which is characterized as very aggressive and associated with poor prognosis and a higher death rate compared to the other molecular subtypes [3]. The process of subtyping breast cancer based on gene expression patterns has clarified differences in biological behavior between subgroups, allowing individualized treatment and better prognosis for each subtype [4].

Sirtuins (SIRT) are NAD+-dependent class-III histone deacetylases, a highly-conserved gene family from yeast to mammals that have drawn increasing attention in recent years due to their action in various pathophysiological processes. In mammals, there are seven known SIRT homologs that localize to different subcellular compartments, and they primarily possess histone deacetylase activity (SIRT1, SIRT2, SIRT3 and SIRT5) or monoribosyltransferase activity (SIRT4 and SIRT6). These sirtuin isoforms can alter a wide variety of substrates involved in cell differentiation, viability, senescence, inflammation, and cellular survival, and thus control diverse key functions ranging from cellular survival to chromatin remodeling. Sirtuins are also closely involved in aging process, lifespan, and various pathologies including cancer, inflammation, immune dysfunction, cardiovascular disorders and neurodegeneration [5, 6].

Silent mating type information regulation 2 homolog 1 (SIRT1), the mammalian counterpart of yeast silent information regulator 2 (Sir2), is the most extensively studied protein in the SIRT family. SIRT1 is involved in key cellular processes such as apoptosis, DNA repair, chromatin remodeling and cancer development [7, 8], but its role in carcinogenesis is controversial, as it can have both tumor-suppressive and tumor-promoting functions, mainly depending on cancer type [9]. For instance, SIRT1-mediated deacetylation of the tumor suppressors p53 [10] and p73 [11] inactivates them, preventing cellular growth arrest, senescence and apoptosis, hence exerting oncogenic functions. On the other hand, SIRT1 is also reported to mediate BRCA1 signaling and inhibit tumor growth through downregulation of oncogenes or by repressing the activity of oncoproteins such as β-catenin [12] and survivin [13]. Furthermore, knockout mice models of SIRT1 are prone to tumor development, which points to a tumor-suppressive SIRT1 action [13]. These seemingly opposite functions might reflect a highly context-specific role of SIRT1 as a tumor-suppressor versus tumor-promotor.

The clinical significance of sirtuins in various human cancers has mostly been evaluated based on sirtuin expression patterns in tumors and non-tumor samples. Generally, overexpression of a protein in tumors indicates its oncogenic properties, whereas reduced expression of a protein indicates its tumor-suppressive properties. Studies using this approach report that SIRT1 is upregulated in a spectrum of cancers including, but not limited to, liver cancer [14], acute myeloid leukemia [15], bone cancer [16], thyroid cancer [17] and skin cancer [18], but downregulated in other cancers including colon cancer [12], oral squamous cell carcinoma [19], glioblastoma and ovarian cancer [20]. Studies in breast cancer have confirmed that SIRT1 is involved in tumorigenesis, metastasis [21] and chemoresistance [22]. However, there have been relatively few studies investigating SIRT1 expression levels to identify its function, and the results are contradictory. A limitation of these studies is that they did not take into account the heterogeneity of various intrinsic breast cancer subtypes, and most of them did not use tissue samples from breast cancer patients but relied on breast cancer cell lines instead. Here, we evaluated both the mRNA and protein expression patterns of SIRT1 using human breast tumors and their corresponding normal breast tissues, in all 5 molecular subtypes of breast cancer. This research brings key insight to the ongoing controversy of SIRT1 behavior in breast cancer carcinoma.

## RESULTS

### Study population characteristics

The breast cancer molecular subtypes studied here spanned luminal A (*n* = 10, 20%), luminal B (HER2-) (*n =* 10, 20%), luminal B (HER2+) (*n =* 10, 20%), HER2-enriched (*n =* 10, 20%) and triple-negative (*n =* 10, 20%). All patients were females aged 45 to 82 years (mean 63.8 ± SD 7.1). Tumor size ranged from 0.5 to 7 cm (2.3 ± 0.5). All tumors were graded according to the modified Scarff-Bloom-Richardson grading system (SBR) as grade 1 (*n =* 3), grade 2 (*n =* 25) and grade 3 (*n =* 22). Samples were ER-, PR- and HER2-positive in *n =* 30 (60%), *n =* 16 (32%) and *n =* 20 (40%) patients, respectively. Table 1 gives the clinico-pathological variables of the 50 breast cancer patients.

**Table 1:** Clinico-pathological characteristics of the breast cancer patients included in this study

	Total	Luminal A	Luminal B (HER2−)	Luminal B (HER2+)	HER2-enriched	Triple- negative	*P* value
Patients, *n* (%)	*N* = 50 (100%)	*n* = 10 (20%)	*n* = 10 (20%)	*n* = 10 (20%)	*n* = 10 (20%)	*n* = 10 (20%)	
Age							0.809
45–65	25 (50)	6 (60)	4 (40)	5 (50)	6 (60)	4 (40)	
>65	25 (50)	4 (40)	6 (60)	5 (50)	4 (40)	6 (60)	
SBR grade							**0.001**
I	3 (6)	3 (30)	0	0	0	0	
II	25 (50)	7 (70)	8 (80)	4 (40)	3 (30)	3 (30)	
III	22 (44)	0	2 (20)	6 (60)	7 (70)	7 (70)	
Size (cm)							0.265
<1.5	10 (20)	2 (20)	3 (30)	2 (20)	1 (10)	2 (20)	
1.5–2.5	21 (42)	7 (70)	4 (40)	5 (50)	2 (20)	3 (30)	
>2.5	19 (38)	1 (10)	3 (30)	3 (30)	7 (70)	5 (50)	
ER							**0.0001**
Positive	30 (60)	10 (100)	10 (100)	10 (100)	0	0	
Negative	20 (40)	0	0	0	10 (100)	10 (100)	
PR							**0.0001**
0%–50%	5 (10)	1 (10)	2 (20)	2 (20)	0	0	
51%–100%	11 (22)	9 (90)	2 (20)	0	0	0	
Negative	34 (68)	0	6 (60)	8 (80)	10 (100)	10 (100)	
HER2							**0.0001**
Positive	20 (40)	0	0	10 (100)	10 (100)	0	
Negative	30 (60)	10 (100)	10 (100)	0	0	10 (100)	
Ki-67							**0.0001**
≤20%	19 (38)	10 (100)	2 (20)	3 (30)	2 (20)	2 (20)	
>20%	31 (62)	0	8 (80)	7 (70)	8 (80)	8 (80)	

ER: Estrogen Receptor, PR: Progesterone Receptor, HER2: Human Epidermal growth factor Receptor 2, Ki-67: cellular marker for proliferation.

### SIRT1 is upregulated in (HRBC) and (H2BC) subtypes and downregulated in (TNBC) subtype

**Figure 1:**
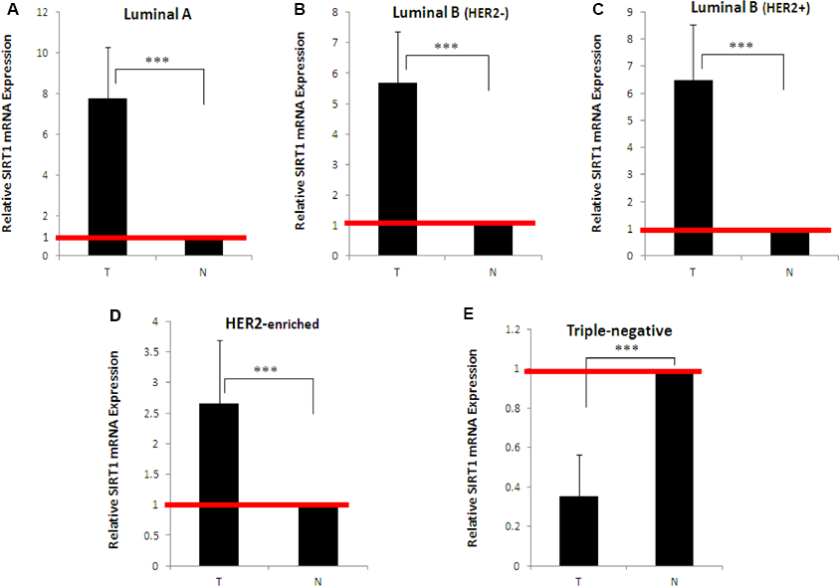
Quantitative expression levels of SIRT1 in different breast tumor subtypes and their matched normal tissue samples SIRT1 expression levels were quantified by real-time quantitative PCR using mRNA extracted from (**A**) *n =* 10 luminal A, (**B**) *n =* 10 luminal B (HER2−), (**C**) *n =* 10 luminal B (HER2+), (**D**) *n =* 10 HER2-enriched, (**E**) *n =* 10 triple-negative breast tumors, and their adjacent normal tissues. SIRT1 mRNA expression was normalized against 18S rRNA levels. SIRT1 expression in breast tumors was expressed as fold-change compared to normal breast tissues (defined as 1). Each real-time PCR reaction was performed in triplicate, the results are expressed as mean ± SD, *P* values were two-tailed and ^***^P < 0.001 was considered statistically significant. T: Tumor, N: Normal.

To assess SIRT1 expression at transcriptional/post-transcriptional level, SIRT1 messenger RNA (mRNA) was extracted from *N =* 50 tumors and their matched normal tissues (*n =* 10 for each of the 5 molecular subtypes), reverse-transcribed into complementary DNA (cDNA), then quantified by real-time quantitative PCR (RT-qPCR). Compared to matched normal tissues, relative SIRT1 mRNA expression was significantly higher in luminal A (mean 7.8 ± SD 2.5, *p* < 0.001; Figure 1A), luminal B (HER2−) (5.7 ± 1.7, *p* < 0.001; Figure 1B), luminal B (HER2+) (6.5 ± 2.1, *p* < 0.001; Figure 1C) and HER2−enriched (2.7 ± 1, *p* < 0.001; Figure 1D), but significantly lower in the triple-negative subtype (0.35 ± 0.2, *p* < 0.001; Figure 1E).

### Positive correlation between SIRT1 expression and the St Gallen molecular classification

**Figure 2:**
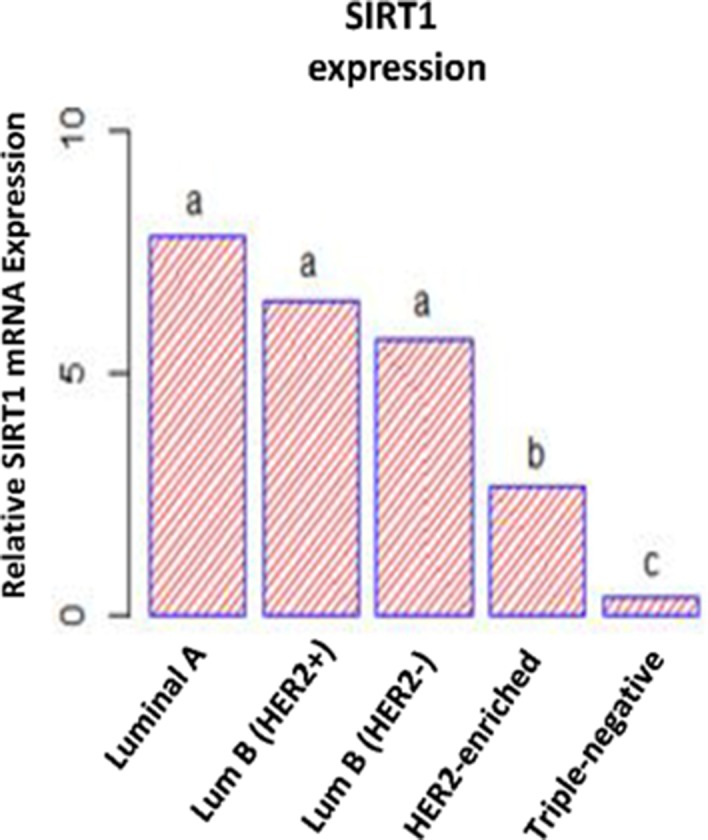
Differential SIRT1 mRNA expression patterns in breast tumors ANOVA followed by Tukey’s multiple comparison test performed on SIRT1 mRNA expression levels. This statistical analysis discerned 3 different SIRT1 expression patterns. The letters ‘a’, ‘b’ and ‘c’ indicated statistical significance between groups.

The differences between SIRT1 mRNA expression levels among the 5 molecular subtypes were further investigated using multi-way analysis of variance (ANOVA) followed by post-hoc analysis. Tukey’s range test was then used for multiple comparisons among mean SIRT1 mRNA expression levels. The statistical procedures distinguished 3 distinct patterns of SIRT1 expression in human breast cancer tumors that correspond to the 3 molecular subtypes: overexpression in (HRBC) subtypes, slight overexpression in the (H2BC) subtype, and underexpression in the (TNBC) subtype (Figure 2).

### Consistency between SIRT1 mRNA and protein expression patterns

**Figure 3:**
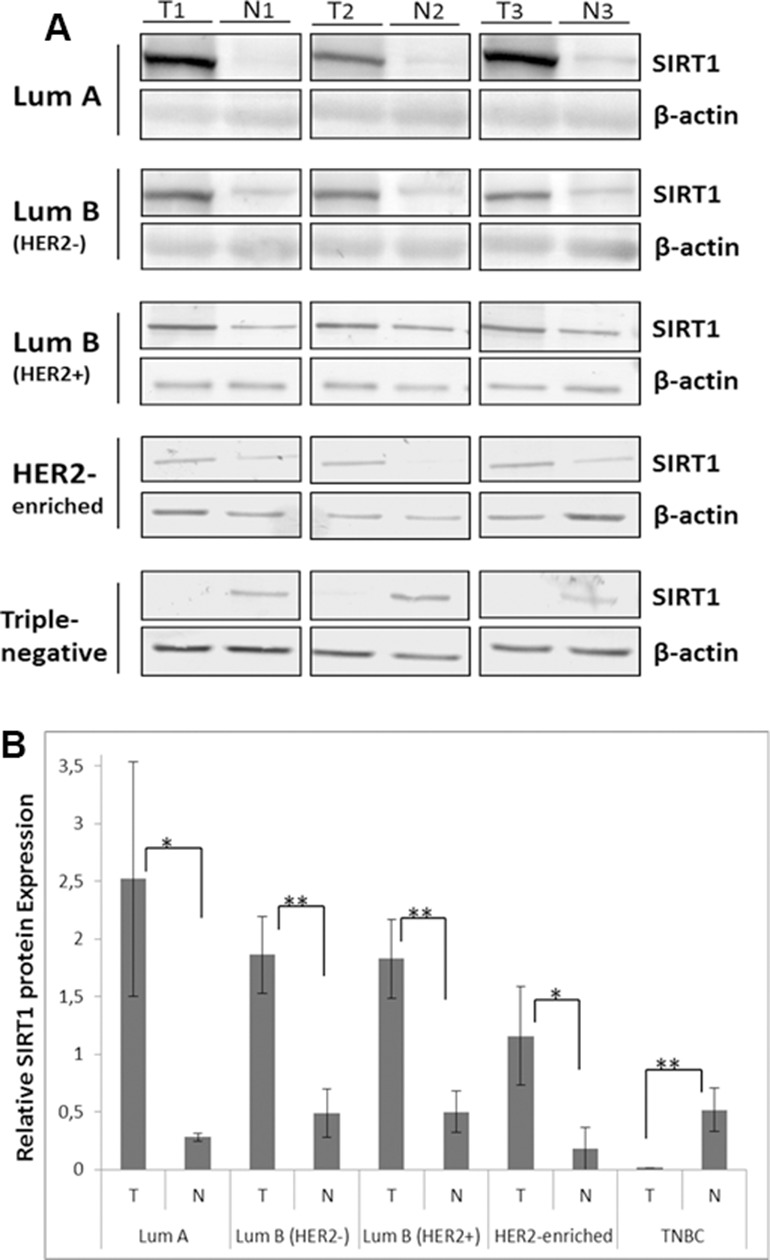
Differential SIRT1 protein expression patterns in breast tumors (**A**) Representative immunoblots of 3 independent experiments showing SIRT1 protein expression in the 5 molecular subtypes of breast cancer. Equal amounts of proteins were immunoblotted with SIRT1 antibody (110 kDa). β-actin (42 kDa) served as loading control. (**B**) Relative SIRT1 protein expression was evaluated using Quantity One software with SIRT1 expression normalized against β-actin as loading control. Each bar represents the mean ± SD of 3 replicate experiments. For the statistical analysis, *P*-values were two tailed, ^*^P < 0.05 and ^*^P < 0.01 were considered statistically significant. T: Tumor, N: Normal.

In order to determine whether SIRT1 transcription levels are equally translated into functional proteins, SIRT1 protein levels were assessed in breast tumors and their matched normal tissue samples using immunoblot analysis. We found that SIRT1 protein expression pattern differs amongst the 5 molecular subtypes, as shown in (Figure 3A). In comparison with normal breast tissue, SIRT1 protein expression was significantly higher in (HRBC) subtypes and in the (H2BC) subtype, but significantly reduced in the (TNBC) subtype (Figure 3B). These results are consistent with the mRNA expression level data.

## DISCUSSION

In order to clarify the biological behavior of SIRT1 and evaluate its role in breast carcinoma, we evaluated SIRT1 expression patterns at the transcriptional and translational levels in human breast tumors and their corresponding normal breast tissues, according to St Gallen molecular subtype class. Although some studies have investigated SIRT1 expression in breast cancer, however, this is the first study to extensively examine SIRT1 mRNA and protein expression levels according to intrinsic subtypes with a sample size that satisfies statistical power requirements.

SIRT1 is a class-III histone deacetylase critically involved in the occurrence and development of a multitude of tumors, and reported to be involved in regulating a multitude of biological processes including apoptosis, cell survival, proliferation and stress response. SIRT1 expression levels have been extensively investigated in many malignancies in order to assess its role. SIRT1 expression and function are found to vary drastically depending on cell and tumor types, making it a multifaceted enzyme with contradictory functions depending on its upstream regulators and downstream targets [23]. SIRT1 overexpression has been reported in several human cancers, it was generally associated with poor prognosis and poor overall survival [24], whereas reduced SIRT1 expression was consistent with a tumor-suppressor role [12, 20].

Several studies have investigated SIRT1 expression in breast cancer, but while some studies found upregulated SIRT1 expression, others did not concur. There are multiple reasons that could explain this discrepancy between studies: the fact that SIRT1 expression was evaluated only at transcriptional level [25], or using only breast cancer cell lines [26, 27], and/or using human breast tissue samples but without accounting for the various molecular subtypes [25, 28–30] or without having a statistically sufficient sample size [28, 31]. This unclear picture promoted us to conduct the study here. The results found here revealed different SIRT1 expression patterns among different breast cancer molecular subtypes. We report significant overexpression of SIRT1 mRNA and protein levels in HRBC and H2BC subtypes, and a significant underexpression in the TNBC subtype. This dual expression pattern of SIRT1 in tumors points to a differential role of SIRT1 in human breast cancer. Based on its expression patterns, SIRT1 most probably has an oncogenic role in the HRBC and H2BC subtypes, in line with Elangovan *et al.* [32] and Ma *et al.* [33], who reported that SIRT1 overexpression in luminal breast cancer subtypes is correlated with an oncogenic behavior. In contrast, SIRT1 may play a tumor-suppressor role in the TNBC subtype, in line with Yi *et al.* [34] who reported that the activation of SIRT1 by a SIRT1-specific activator YK-3-237 induced deacetylation of the mutant form of p53 (mtp53), suppressing the proliferation and arresting the cell growth of triple-negative breast cancer cell lines. Furthermore, Simic *et al.* [35] showed that ectopic expression of SIRT1 suppresses cancer metastasis and tumor cell invasion. Moreover, our findings showed a positive correlation between SIRT1 expression and St Gallen molecular subtype classification. After classifying the breast tumors used in ascending order of aggressivity, decreased SIRT1 expression was found to correlate with increased breast cancer aggressivity and poor prognosis. We conclude that SIRT1 may serve as a prognostic biomarker in breast cancer carcinomas.

In conclusion, this study demonstrated for the first time a differential pattern of SIRT1 expression in breast cancer at both transcriptional and protein level using human breast tumors and their uninvolved benign counterparts, it also established an association between SIRT1 expression and St Gallen classification. Taken together, these results suggest that SIRT1 plays a bivalent subtype-dependent role in breast carcinoma, and that SIRT1 could also be a potential prognostic marker in breast cancer. Given that SIRT1 regulates a wide range of substrates directly involved in the tumorigenesis process, it could make a novel and potentially promising anticancer therapeutic target, especially if results from clinical trials currently testing specific SIRT1 inhibitors are deemed good.

## MATERIALS AND METHODS

### Study population selection and collection of tissue samples

This study included a total of 50 patients admitted to the Centre Jean Perrin from October 2012 to September 2016 for cancer treatment, and diagnosed with breast cancer carcinoma. Patients were informed about the study and gave informed consent prior to inclusion. All 50 tumors and their adjacent normal breast tissues came from the Centre Jean Perrin Biological Resource Center, where they were put in cryotubes and stored in liquid nitrogen at −196°C. Patients who received chemotherapy, hormonal therapy and/or radiotherapy for cancer in other parts of the body were excluded from the study, as were patients with predisposition to breast cancer and/or family members with breast cancer.

### Intrinsic breast cancer subtype classification

The breast carcinomas were classified into 5 molecular subtypes according to St Gallen breast cancer conference guidelines [2] based on estrogen receptor (ER), progesterone receptor (PR), human epidermal growth factor receptor 2 (HER2), and Ki-67 proliferative index, as follows:

Luminal A: [ER- and/or PR-positive, HER2-negative, and Ki-67 <14%]

Luminal B (HER2-): [ER- and/or PR positive, HER2-negative and Ki-67 ≥14%]

Luminal B (HER2+): [ER- and/or PR-positive, HER2-positive, and any Ki-67]; these 3 subtypes are included in the hormone receptor-positive breast cancer (HRBC) group.

HER2-enriched/HER2 breast cancer (H2BC): [ER- and/or PR-negative, HER2 overexpressed]

Triple-negative breast cancer (TNBC): [ER-, PR-, and HER2-negative].

### Total RNA isolation from tissues and reverse transcription (RT)

Tumoral and non-tumoral tissue samples were cut into pieces and homogenized with TissueRuptor^®^ (Qiagen, Hilden, Germany). Total RNA was isolated using TRIzol Reagent (Ambion, Life Technologies, CA) then extracted using a PureLink RNA Mini Kit (Invitrogen, Thermo Fisher Scientific, CA). RNA samples purity was verified using NanoDrop ND-8000 spectrophotometer. cDNA was then obtained using the high-capacity cDNA reverse transcription kit (AB Applied Biosystems, Foster City, CA) according to the manufacturer’s protocol.

### RT-qPCR methods and data analysis

Synthesized cDNA was amplified using TaqMan Gene expression PCR Master Mix (AB Applied Biosystems) as per the manufacturer’s protocol. Each duplex PCR was assembled using 96-well MicroAmp Optical plates (AB Applied Biosystems) with 25 ng of template cDNA in a total volume of 25 µL containing 12.5 µL TaqMan Gene Expression Master Mix (2X), 1.25 µL TaqMan Gene Expression assay-on-demand SIRT1 [Hs01009006_m1] (200 nM), 0.25 µL endogenous control 18S rRNA primers (10 µM) and 0.25 µL 18S rRNA probe (5 µM). Primer sets for specific reverse transcription of SIRT1 and endogenous control 18S rRNA were all obtained from (AB Applied Biosystems), and are as follows: SIRT1 forward 5-CCTGTGAAAGTGATGAGGAGGATAG-3; reverse 5-TTGGATTCCCGCAACCTG-3. 18S forward: 5′-CGG CTA CCA CAT CCA AGG AA-3′, reverse: 5′-GCT GGA ATT ACC GCG GCT-3′, probe: 5′-TGCTGG CAC CAG ACT TGC CCT C-3′. The thermal reaction cycles used were 50°C for 2 min, 95°C for 10 min, and 40 cycles of 95°C for 15 sec and 60°C for 1 min. The signal was collected at the endpoint of each cycle using an AB Prism 7900 Sequence Detector System (AB Applied Biosystems). Relative gene expression was determined by normalizing to reference gene *18S* and according to the relative quantitative (ΔΔCt) method. Fold change in SIRT1 expression was then calculated using the (2−ΔΔCt) method. SIRT1 mRNA expression in breast tumors was calculated relative to the matched normal breast tissues. All experiments were done in triplicate, and results were expressed as means ± SD.

### Protein extraction and immunoblot analysis

Frozen tissues were homogenized before being lysed using T-PER™ Tissue Protein Extraction Reagent (ThermoFisher Scientific) containing protease inhibitor cocktail (Sigma Aldrich). Whole protein extracts were resolved by electrophoresis on 8% sodium dodecyl sulfate polyacrylamide gel (SDS-PAGE), then electro-transferred onto polyvinylidene difluoride membranes (Immobilon-P, PVDF, 0.45 µm, Merck Millipore) in transfer buffer (25 mM Tris-HCL (pH 7.6), 192 mM glycine, 10% methanol). The membranes were blocked with 5% non-fat milk in 0.1% TBS-tween and later immunoblotted with monoclonal anti-SIRT1 antibody (1/500, MAb-063-050, Diagenode) or monoclonal anti-β-actin antibody (1/5000, CP01, Merck Millipore). Membranes were then washed and incubated with alkaline phosphatase-conjugated secondary antibody anti-mouse IgG (1/2000, S3721, Promega). Immunolabeling was detected using Western Blue^®^ Stabilized substrate for Alkaline Phosphatase (Promega) at room temperature.

### Statistical analysis

Correlation between the clinical parameters of our study groups were examined by chi-square test (χ^2^ test) using SPSS statistics software (SPSS Inc., Chicago, IL). Multiple-group comparisons were performed by ANOVA using R software (version 3.0.3). Post-hoc comparison of the means was performed using Tukey’s multiple comparison test when the F-test was significant (*p* < 0.05). Relative expression levels of SIRT1 protein assayed by immunoblotting were assessed numerically using Quantity One software (Bio-Rad, CA). Groups were compared using a two-tailed unpaired Student’s *t*-test carried out after a Fisher’s exact test. All experiments were done in triplicate and the results were expressed as mean ± SD. In all cases, statistical significance was set at the following *P*-values: ^*^P < 0.05, ^**^P < 0.01 and ^***^P < 0.001.
